# Using graph theory as a common language to combine neural structure and function in models of healthy cognitive performance

**DOI:** 10.1002/hbm.26258

**Published:** 2023-03-07

**Authors:** Marta Czime Litwińczuk, Nils Muhlert, Nelson Trujillo‐Barreto, Anna Woollams

**Affiliations:** ^1^ Division of Neuroscience and Experimental Psychology University of Manchester Manchester UK

**Keywords:** adult, cognition, functional connectivity, graph theory, multimodal, multivariate, structural connectivity

## Abstract

Graph theory has been used in cognitive neuroscience to understand how organisational properties of structural and functional brain networks relate to cognitive function. Graph theory may bridge the gap in integration of structural and functional connectivity by introducing common measures of network characteristics. However, the explanatory and predictive value of combined structural and functional graph theory have not been investigated in modelling of cognitive performance of healthy adults. In this work, a Principal Component Regression approach with embedded Step‐Wise Regression was used to fit multiple regression models of Executive Function, Self‐regulation, Language, Encoding and Sequence Processing with a collection of 20 different graph theoretic measures of structural and functional network organisation used as regressors. The predictive ability of graph theory‐based models was compared to that of connectivity‐based models. The present work shows that using combinations of graph theory metrics to predict cognition in healthy populations does not produce a consistent benefit relative to making predictions based on structural and functional connectivity values directly.

## Graphical abstract

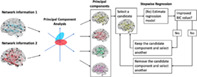

## 1 INTRODUCTION

In statistical modelling, complex social, physical, economic and biological systems can be represented by graphs. A graph is a set of elements, referred to as nodes, and connections between them, referred to as edges. An adjacency matrix is a square matrix representing the graph. This approach to characterising complex systems has become increasingly popular in neuroscience as human brains naturally form networks that can be represented by graphs (Bassett et al., 2020; Fornito et al., 2016; Sporns et al., 2005). For example, brains are physically made up of neuronal populations that can constitute nodes and white matter connections between them that can constitute edges (structural connectivity [SC]) (Sporns et al., 2005). In addition, activity in disparate remote neuronal populations can act as nodes and the coordination of their activity can be considered as edges (functional connectivity [FC]) (Bullmore & Sporns, 2009; Friston, 2002). These graph representations of the brain can then be used to try and better understand the relationship between brain structure and function. In this study, we consider what are the benefits of using the graph theory approach to combine SC and FC.

Analyses of brain connectivity have demonstrated an association between the strength of SC and FC. Evidence demonstrates that white matter tends to connect neuronal populations that show synchronised activation patterns (Greicius et al., 2008; Johansen‐Berg et al., 2004; Jung et al., 2017; Vázquez‐Rodríguez et al., 2019). The strength of SC has also been shown to correlate with FC (Honey et al., 2009; Koch et al., 2002; Skudlarski et al., 2008), and to predict patterns of FC with moderate accuracy (Honey et al., 2009; Honey et al., 2010). Furthermore, evidence demonstrates moderate coupling of age‐related changes of SC and FC strength across lifespan (Baum et al., 2020; Romero‐Garcia et al., 2014). Similarly, coupling of SC and FC abnormalities have been observed in neurologically atypical populations such as patients with autism spectrum disorder (Delmonte et al., 2013; Park et al., 2021), epilepsy (Chiang et al., 2015), schizophrenia (Cocchi et al., 2014), depression (Jiang et al., 2019), multiple sclerosis (Kulik et al., 2022) and Alzheimer's disease (Zhou et al., 2010).

Interpretation of the relationship between SC and FC is not trivial. SC strength generally reflects the number of diffusion tracking streamlines connecting pairs of regions, and they are generally interpreted as white matter connections. In contrast, FC strength reflects statistical associations between activation amplitude across pairs of regions and these values are generally interpreted as coordination of activity. Consequently, a direct comparison of SC with FC is somewhat similar to the comparison of “apples and oranges”. Furthermore, some characteristics of FC may not be reflected by SC. For example, SC is largely static, changing over long periods (Betzel et al., 2014). Meanwhile, FC is dynamic and shows changes in its topological configuration between cognitive states and shifting environmental pressures (Chang & Glover, 2010; Gonzalez‐Castillo et al., 2015; Mecacci et al., 2004; Park et al., 2012; Shirer et al., 2012). This suggests that edge‐by‐edge divergence between SC and FC may occur due to changing configurations of FC (Park & Friston, 2013). Additionally, a pair of regions may display a strong FC in the absence of direct structural connections (Ashourvan et al., 2019; Friston, 2002; Hagmann et al., 2008; Honey et al., 2009, 2010; Liao et al., 2015; Røge et al., 2017; Sun et al., 2012; Thomas et al., 2009), referred to as indirect FC. This is likely related to the dynamic nature of FC and its ability to produce adaptive responses (Park & Friston, 2013). Indirect FC further exuberates the problem that SC and FC are not directly comparable, but their uniqueness is valid and meaningful. Consequently, SC and FC are likely to capture both shared and unique variance in predicting outcomes.

Some investigations have complemented connectivity analysis by obtaining graph theory measures of network organisation (Rubinov & Sporns, 2010). These measures quantitatively describe the architecture of networks. In neuroscience, graph theory measures are obtained after raw connectivity matrices have been calculated. After SC and FC have been additionally processed in this manner, their organisation can be directly compared. This procedure sidesteps the problem that the values of SC and FC have different interpretations because graph theory measures express the same information across networks. As a result, we can make direct comparisons between SC and FC and then meaningfully interpret how their organisation differs. For example, neuroscience has focused on comparing the balance between segregation and integration of information within each connectivity (Park et al., 2008). Park and colleagues have demonstrated that both SC and FC balance segregation and integration of information processing. This balance was estimated with analysis of each connectivity's tendency to produce clusters of strongly connected nodes relative to its tendency to produce short paths between pairs of nodes (aka small‐world architecture). However, SC had greater global and local efficiency, as measured by the length of shortest paths between pairs of nodes. Meanwhile, FC had greater assortativity, as measured by the network tendency to link pairs of nodes that have a similar amount of connections with the rest of the network. Park and colleagues argued that this suggests that SC is more efficiently wired than FC, serving as a scaffold for FC. Furthermore, they proposed that with greater assortativity FC is overall more suited for supporting a variety of sensory and cognitive tasks and it is more resilient to node damage than SC.

Graph theory can also be used to focus on the study of local patterns of edges to explore how nodes are embedded within the SC and FC. Several studies have meaningfully related SC and FC using various local metrics and found a degree of shared organisational patterns (Battiston et al., 2017; Bullmore & Sporns, 2012; Goñi et al., 2013; Park et al., 2008). For example, one specific local measure is the rich club coefficient, which measures the extent to which well‐connected nodes also connect with each other (van den Heuvel & Sporns, 2011). Grayson et al. (2014) have demonstrated that the same nodes can be classified as belonging to rich club across SC and FC. This is important because rich club architecture supports efficient information exchange across sections of the brain (van den Heuvel & Sporns, 2013), which suggests a shared role in network communication of specific regions across SC and FC. Therefore, using local graph theory measures in network analysis yields a common reference point that allows for a meaningful interpretation of the relationship between SC and FC.

Previous work has utilised raw connectivity of SC and FC to predict cognition (Dhamala et al., 2021; Litwińczuk et al., 2022; Rasero et al., 2021). Raw connectivity contains information about each edge of the network. For example, for SC this can reflect the number of streamlines connecting each pair of regions. Meanwhile, for FC this can be the temporal correlation between the activities of each pair of regions indicating the strength of their statistical associations. This means that connectivity matrices are rich in information but they may also be prone to inclusion of noise, which might affect the accuracy of predictive models of cognition based on raw connectivity. In contrast, graph theory measures characterise the organisation of the network (either structural or functional) in different respects, depending on the specific measures used. While these summarisations may reduce the amount of noise in the model predictors by focussing on specific aspects of the organisation of the network, they also disregard connectivity information that might be relevant to predict cognition. A potential advantage of graph theory versus raw connectivity to predict cognition is that the measures of network organisation produced by the former have the same meaning across both structural and functional networks. Thus, graph theory may serve as a common language or an effective translation tool between SC and FC, which might facilitate the interpretation of models combining SC and FC. This can help understand why a combination of SC and FC would benefit only specific cognitive domains (Dhamala et al., 2021; Litwińczuk et al., 2022; Rasero et al., 2021). However, to‐date no study has used graph theory measures to predict cognitive skill in healthy individuals (Farahani et al., 2019). Thus, it is unknown if graph theory measures could be effectively implemented in predictive modelling of cognition. If this is the case, then it is unknown how the resulting graph theory predictive models compare to predictive models composed with raw connectivity.

To expand on the research from previous predictive modelling studies, the present work aims to investigate if graph theory measures of network organisation (e.g., clustering coefficient, node degree, small world coefficient, etc.) can be used to construct predictive models of healthy adult cognition. Furthermore, our work aims to investigate if these predictive models are better at predicting cognition than predictive models constructed with raw connectivity (i.e., white matter connections and statistical associations in activation across regions). To achieve this, 19 graph theory measures were obtained. These measures were then used to fit predictive models of cognition. Then, we compared the effectiveness of graph theory‐based predictive models in predicting cognitive performance with effectiveness of connectivity‐based predictive models obtained in our previous work (Litwińczuk et al., 2022). We tested the hypothesis that predictive models constructed with any graph theory measures will offer better predictions of cognition than predictive models constructed with raw connectivity. Codes used to implement the analysis are available on GitHub (https://github.com/MCLit/GT/PCA-SWR).

## 2 METHODS

### 2.1 Participants

Neuroimaging and cognitive data were obtained for 250 unrelated subjects from the 1200‐subject release of the Human Connectome Project (HCP). For consistent treatment of behavioural and neuroimaging subjects' data selection, one subject was excluded from the neuroimaging analysis due to incomplete behavioural data. The sample consisted of 138 females and 111 males in the age range between of 22 and 36 years.

### 2.2 Measures of cognition

The present work used the measures of cognition from our previous work (Litwińczuk et al., 2022). Briefly, Principal Component Analysis (PCA) with VARIMAX rotation was used as a feature extraction method from the behavioural dataset. Analysed tasks included: Picture Sequence Memory, Dimensional Change Card Sort, Flanker Inhibitory Control and Attention Task, Penn Progressive Matrices, Oral Reading Recognition, Picture Vocabulary, Pattern Comparison Processing Speed, Delay Discounting, Variable Short Penn Line Orientation Test, Short Penn Continuous Performance Test, Penn Word Memory Test, and List Sorting. These assessments were obtained from the Blueprint for Neuroscience Research–funded NIH Toolbox for Assessment of Neurological and Behavioral function (http://www.nihtoolbox.org) and tasks from the Penn computerised neurocognitive battery (Gur et al., 2010). The extracted PCA rotated components reflected specific latent cognitive domains, interpreted as Executive Function, Self‐regulation, Language, Encoding and Sequence Processing. The present work uses the PCA scores obtained previously for each cognitive domain.

### 2.3 Minimally processed neuroimaging data

The HCP provides minimally processed neuroimaging data that were used here, the data acquisition and processing pipeline has been discussed in detail by Glasser et al. (2013). All neuroimaging data were collected with a 3 T Siemens “Connectome Skyra” scanner that uses the Siemens 32‐channel RF receive head coil and with an SC72 gradient insert (Ugurbil et al., 2013). Here, we utilised Version 3 of the minimal processing pipeline implemented with FSL 5.0.6 (Jenkinson et al., 2012) and FreeSurfer 5.3.0‐HCP (Dale et al., 1999).

T1 weighted MR images were acquired with a 3D MPRAGE sequence (TR = 2400 ms, TE = 2.14, TI = 1000 ms, flip angle = 8°, FOV = 224 by 224 mm, voxel size = 0.7 mm isotropic). rs‐fMRI data were collected using the gradient‐echo EPI (TR = 720 ms, TE = 33.1 ms, flip angle = 52°, FOV = 208 by 180 mm, 70 slices, thickness = 2.0 mm, size = 2.0 mm isotropic). Scans were collected in two sessions, each lasting approximately 15 min. The rs‐fMRI data were collected both in left‐to‐right and right‐to‐left directions. In addition, in the original data, spin echo phase reversed images were acquired for registration with T1 images and the spin echo field maps were acquired for bias field correction. Diffusion weighted MR images were acquired with spin‐echo EPI sequence (TR = 5520 ms, TE = 89.5 ms, flip angle = 78°, refocusing flip angle = 160°, FOV = 210 by 180 mm, 111 slices, thickness = 1.25 mm, size = 1.25 mm isotropic). Each gradient consisted of 90 diffusion weighting directions plus 6 *b* = 0. There were three diffusion weighed shells of *b* = 1000, 2000, and 3000 s/mm^2^. SENSE1 multi‐channel image reconstruction was used (Sotiropoulos et al., 2013).

### 2.4 Additional processing of neuroimaging data

Neuroimaging data were processed following the same processing pipeline as in our previous work (Litwińczuk et al., 2022).

#### 2.4.1 Structural data and structural connectivity calculation

As additional steps to the minimal processing pipeline, the diffusion data were further analysed using the BEDPOSTX procedure in FSL, which runs Markov Chain Monte Carlo sampling to estimate probability distributions on diffusion parameters at each voxel. This information was used in the FDT module of FSL to run ROI‐to‐ROI probabilistic tractography with ProbtrackX. Tractography was run between parcels obtained with a high‐resolution functionally defined brain parcellation with 278 parcels (Shen et al., 2013). During tractography, 5000 streamlines were initiated from each voxel with a step length of 0.5 mm (Behrens et al., 2003, 2007). Streamlines were constrained with a curvature threshold of 0.2, a maximum of 2000 steps per streamline and a volume fraction threshold of subsidiary fibre orientations of 0.01. An SC matrix between regions was constructed by first counting the number of streamlines originating from a seed region i that reached a target region j ($M_{\mathrm{ij}}$). These counts are asymmetric since the count of streamlines from region i to j is not necessarily equal to the count of streamlines from region j to i ($M_{\mathrm{ij}}\neq M_{\mathrm{ji}}$), but they are highly correlated for all subjects (lowest Pearson's Correlation was 0.76, *p* < .001). Based on these counts, the weight $W_{\mathrm{ij}}$ (entries of the SC matrix) between any two pairs of regions i and j was defined as the ratio of the total streamline counts in both directions ($M_{\mathrm{ij}}+M_{\mathrm{ji}}$), to the maximum possible number of streamlines that can be shared between the two regions, which is $\left(N_{i}+N_{j}\right)*5000$ (where $N_{i}$ and $N_{j}$ are the number of seed voxels in regions i and j, respectively):

$$W_{\mathrm{ij}}=\frac{\left(M_{\mathrm{ij}}+M_{\mathrm{ji}}\right)}{\left(N_{i}+N_{j}\right)*5000}$$

Similar to previous studies, the weight $W_{\mathrm{ij}}$ can be interpreted as capturing the connection density (number of streamlines per unit surface) between nodes i and j, which accounts for possible bias due to different sizes of the seed regions (Hagmann et al., 2008; Ingalhalikar et al., 2013). Note that the SC matrix defined based on these weights is symmetric because swapping around the regions' indices does not change the result; and it is also normalised between 0 and 1, because the maximum value of the numerator can only be reached when all streamlines originating from each of region reach the other region, so that $M_{\mathrm{ij}}=N_{i}*5000$ and $M_{\mathrm{ji}}=N_{j}*5000$, which gives $W_{\mathrm{ij}}=1$. Evidence suggests that structural connectivity is most sensitive to individual differences with moderate‐to‐high thresholding (Buchanan et al., 2020) and produces least false positive and negative results (de Reus & van den Heuvel, 2013), therefore an 80% proportional threshold was applied.

#### 2.4.2 Functional data and functional connectivity calculation

The minimally processed images were obtained for rs‐fMRI to compute FC based on pair‐wise correlations (Glasser et al., 2013). Next, the following steps were taken to further process data using the CONN Toolbox (Whitfield‐Gabrieli & Nieto‐Castanon, 2012) with the use of the standard FC processing pipeline (Nieto‐Castanon, 2020). Briefly, images were realigned, slice‐timing correction was conducted, and outlier detection of functional images for scrubbing was performed with Artefact Detection Tools (ART, https://www.nitrc.org/projects/artifact_detect/). Grey matter, white matter, cerebrospinal fluid, and non‐brain tissues were then segmented. Images were normalized and smoothed with a 6 mm Full Width at Half Maximum Gaussian kernel. Next, the data were denoised with default Conn denoising options using the anatomical component‐based noise correction procedure (Behzadi et al., 2007). This procedure removes artefactual components from the data, including noise components from cerebral white matter and cerebrospinal areas, subject‐motion parameters (Friston et al., 1996), identified outlier scans (Power et al., 2014), and constant and first‐order linear session effects (Whitfield‐Gabrieli & Nieto‐Castanon, 2012). Then standard denoising steps were applied including scrubbing, motion regression and application of a high pass filter (0.01 Hz cut‐off), and a low pass filter (0.10 Hz cut‐off).

FC analysis was performed based on the same high‐resolution brain parcellation used in the SC computations (Shen et al., 2013). The average blood oxygenation level‐dependent signal in each ROI was obtained and the pairwise (ROI‐to‐ROI) correlation of the averaged signals was calculated. Since the CONN toolbox produces Fisher's *Z*‐scores (Fisher, 1915), a hyperbolic tangent function was used to reverse the Fisher's transformation, and obtain original correlation values ranging between −1 and 1. Negative correlations were transformed to positive by taking their absolute values and a proportional 80% FC threshold was then applied (Garrison et al., 2015; van den Heuvel et al., 2017).

### 2.5 Graph theory

Graph theoretic measures were calculated based on the weighted, undirected SC and FC matrices of every subject, using The Brain Connectivity Toolbox (http://www.brain-connectivity-toolbox.net). Measures of node, edge and global network organisation are described in Table 1. The clustering coefficient was obtained with Onnela's algorithm (Onnela et al., 2005). Network modules for within module node degree and participation coefficient were defined with Newman's algorithm (Newman, 2006). Shortest path length, used for betweenness centrality, participation coefficient, node eccentricity, and local efficiency, was calculated using the Floyd–Warshall Algorithm applied to the weighted graph obtained from the inverse of each connectivity matrix. Small‐world propensity has been developed by Muldoon et al. (2016).

**TABLE 1:** A summary of graph theory measures obtained within this work.

Node measures	Edge measures	Global measures
Node degree Within module node degree Node strength Clustering coefficient Eigenvector centrality Betweenness centrality Participation coefficient Node eccentricity Local efficiency	Edge betweenness centrality Matching index Path transitivity	Average clustering coefficient Characteristic path length small world propensity Global efficiency Assortativity Modularity statistic Transitivity Size of core resulting from core‐periphery partition

### 2.6 Model construction and model comparisons

**FIGURE 1:**
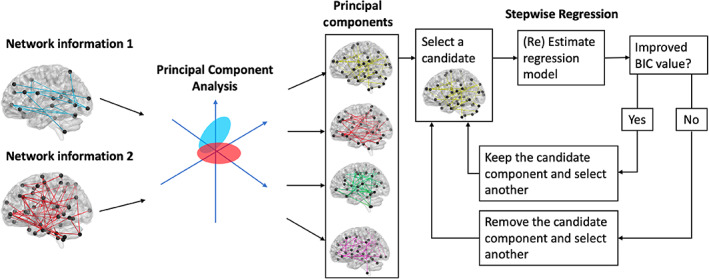
Schematic presenting the Principal Component Regression with Step‐Wise Regression pipeline. Here, network information input to the model can either constitute raw connectivity and/or any number of graph theory measures.

Within this work, we compare the quality of predictive models constructed from raw connectivity with models constructed with graph theory measures of network organisation. All models of cognition were constructed using the Principal Component Regression with Step‐Wise Regression (SWR‐PCR) (Litwińczuk et al., 2022) (Figure 1). In our previous work, the SWR‐PCR pipeline was applied to raw connectivity to produce predictions of cognition. Here, these already fitted models constituted a reference point. In addition, we now applied the SWR‐PCR approach to graph theory measures. This allows for a direct comparison of raw connectivity‐based models with graph theory‐based models to assess the gain (if any) of characterising raw connectivity in terms of graph theory to predict cognition.

During SWR‐PCR, separate linear regression models were fit to each individual cognitive domain (5 domains) using various combinations of graph theory measures of structural and functional networks as predictors. To fit each model we used the Principal Component Regression (PCR) approach, where the predictor's matrix of graph theory measures is first orthogonalised by using PCA to obtain the associated orthogonal principal components' scores. The extracted component scores are then used as candidate predictors in a step‐wise regression (SWR) analysis. SWR was implemented with Bayesian Information Criterion (BIC) as a criterion for including or excluding components (ΔBIC = 0). BIC balances the goodness‐of‐fit of the model (model accuracy) against its complexity (the number of parameters included in the model). Consequently, components were only added to or removed from the regression model if they improved model quality. Finally, the resulting regression coefficients in PCA space were projected back to the original space of graph theoretic measures.

Each graph theoretic measure describes one network property, such as the strength of the node or its degree. Due to their complementary nature, individual graph theory measures can therefore provide a limited explanation of cognition (Appendix S1). The remainder of this work focuses on exploring the complementary nature of the information provided by the graph theoretic measures used to characterise node embedding, edge embedding and global network architecture. For each of the SC, FC and combined connectivity (CC) networks, five separate regression models were fitted using: i) global measures, ii) node measures, iii) edge measures, iv) local measures (i.e., edge and node measures) and v) local and global measures; to predict each cognitive domain. To combine various measures into each one of the five models, individual measures were stacked in a single matrix along the measure's dimension, resulting in a number of subjects by number of measures matrix of predictors.

The Bootstrap Bias Corrected Cross‐Validation (BBC‐CV) was implemented to validate the PCR as a learning method and to evaluate the (out‐of‐sample) predictive performance of the models (Tsamardinos et al., 2018). Permutation (randomisation) testing was used to assess how likely it is to get the observed models' performance by chance. Specifically, the saved predictions during the BBC‐CV were randomised (sampled without replacement) 10,000 times and the models' performance statistics (coefficient of determination) were estimated for each randomisation. This null distribution was then used to assess the observed model performance statistics in the non‐permuted data. That is, a *p*‐value for testing models' performance was determined by computing the proportion of resampled statistics at least as high or greater than the observed statistics. As a complementary analysis, we used the non‐parametric Wilcoxon rank sum tests for equal medians to assess the significance of the difference in performance between different connectivity models and graph theory models. These comparisons were only done for models which performed better than chance, and the results were based on coefficient of determination.

Finally, we compared connectivity‐based models from our previous work (Litwińczuk et al., 2022) with graph theory‐based models obtained here. Model comparison was conducted for each cognitive construct using Bayesian information criterion (BIC) (Schwarz, 1978). That is, the BIC value of the connectivity models was subtracted from the BIC of the graph theory models. Results were then interpreted so that, given any two models $M_{1}$ and $M_{2}$, a positive difference ($\text{ΔBIC}=\mathrm{BIC}\left(M_{1}\right)-\mathrm{BIC}\left(M2\right)$) is interpreted as weak (barely worth a mention) (1–3 units), positive (3–20 units) or strong (20–150 units) evidence in favour of $M_{2}$ (Kass & Raftery, 1995). To complement this analysis, models were further assessed in terms of their coefficients of determination in the sample of 249 participants (Poldrack et al., 2020) (Appendix S2).

## 3 RESULTS

### 3.1 Bayesian information criterion model comparison

**FIGURE 2:**
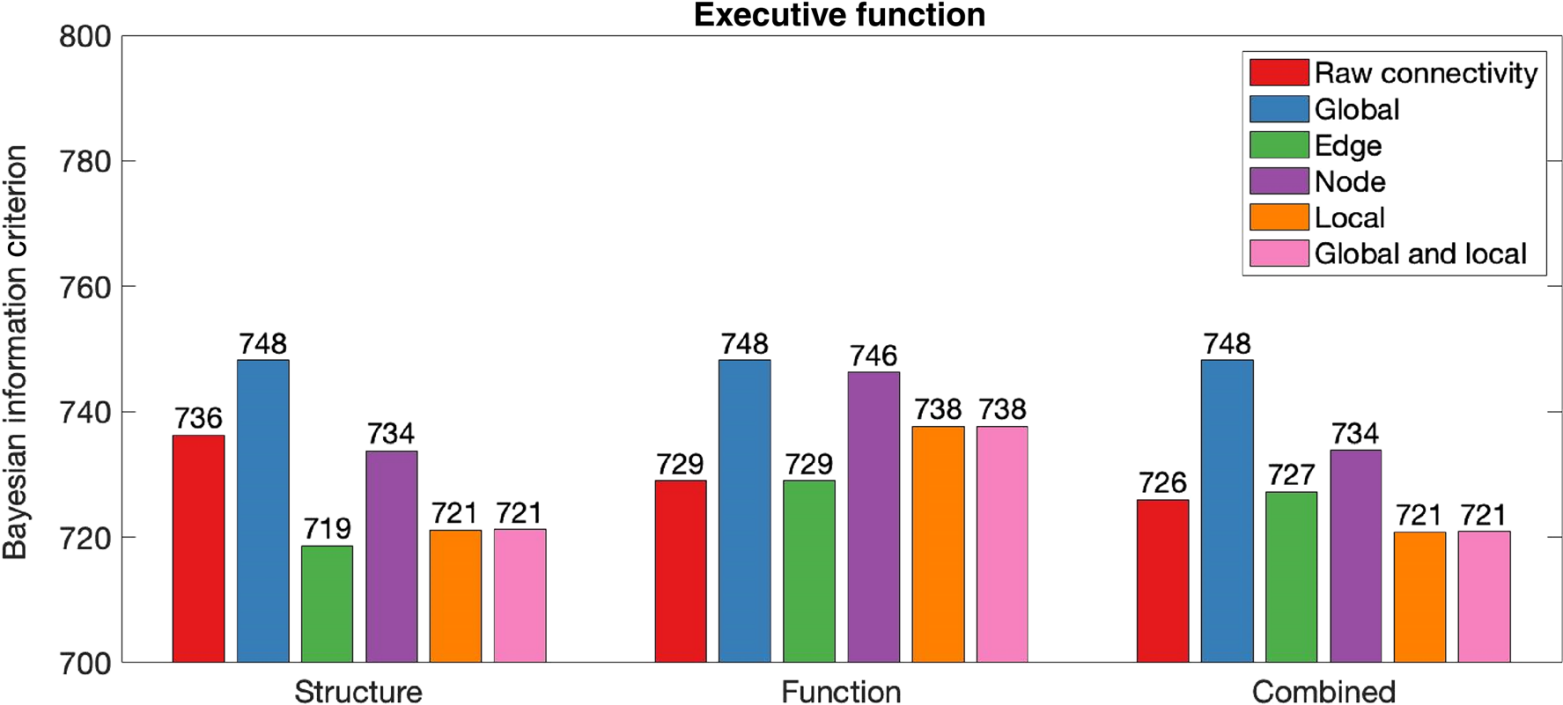
Bayesian information criterion (BIC) model evidence for connectivity and graph theory models of Executive Function. Models with lower BIC values are favoured.

**FIGURE 3:**
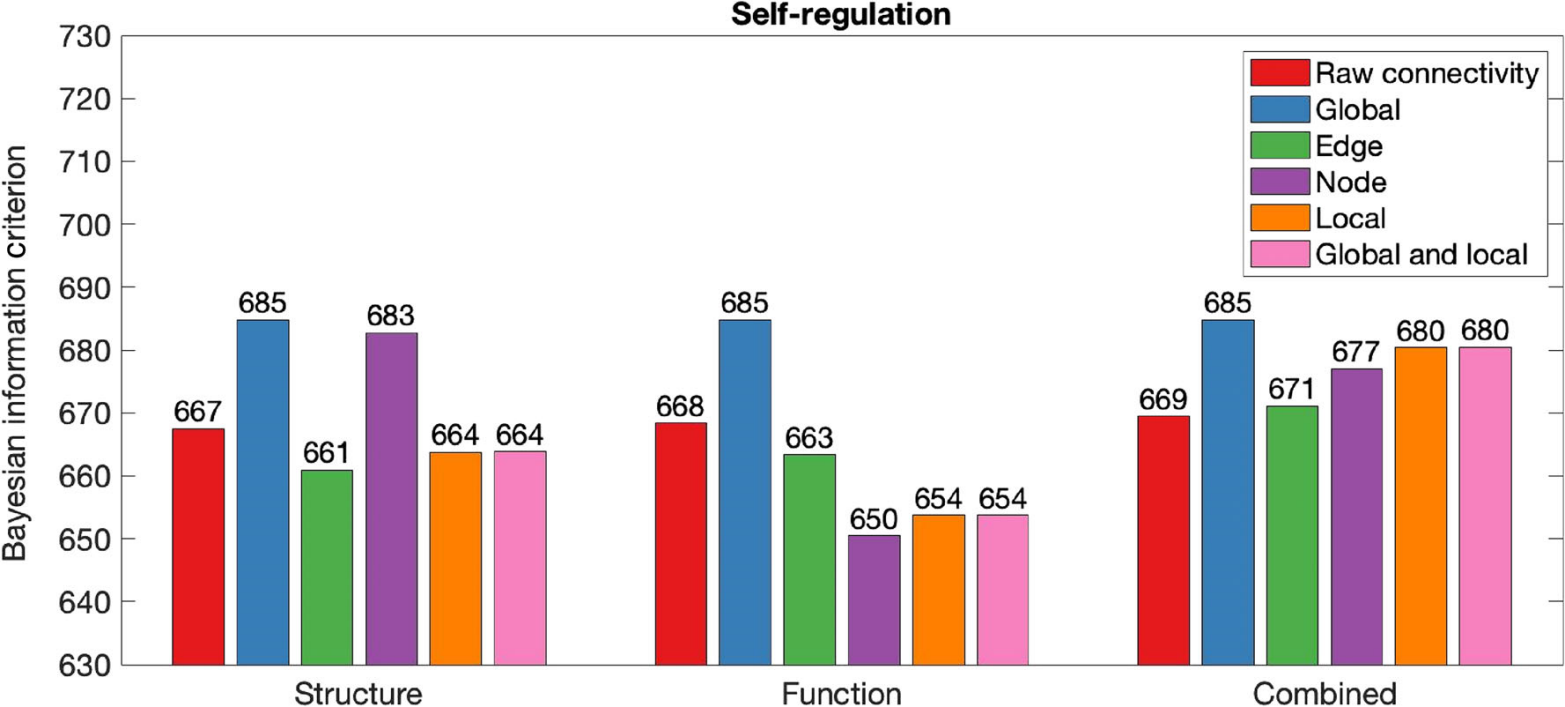
Bayesian information criterion (BIC) model evidence for connectivity and graph theory models of Self‐regulation. Models with lower BIC values are favoured.

**FIGURE 4:**
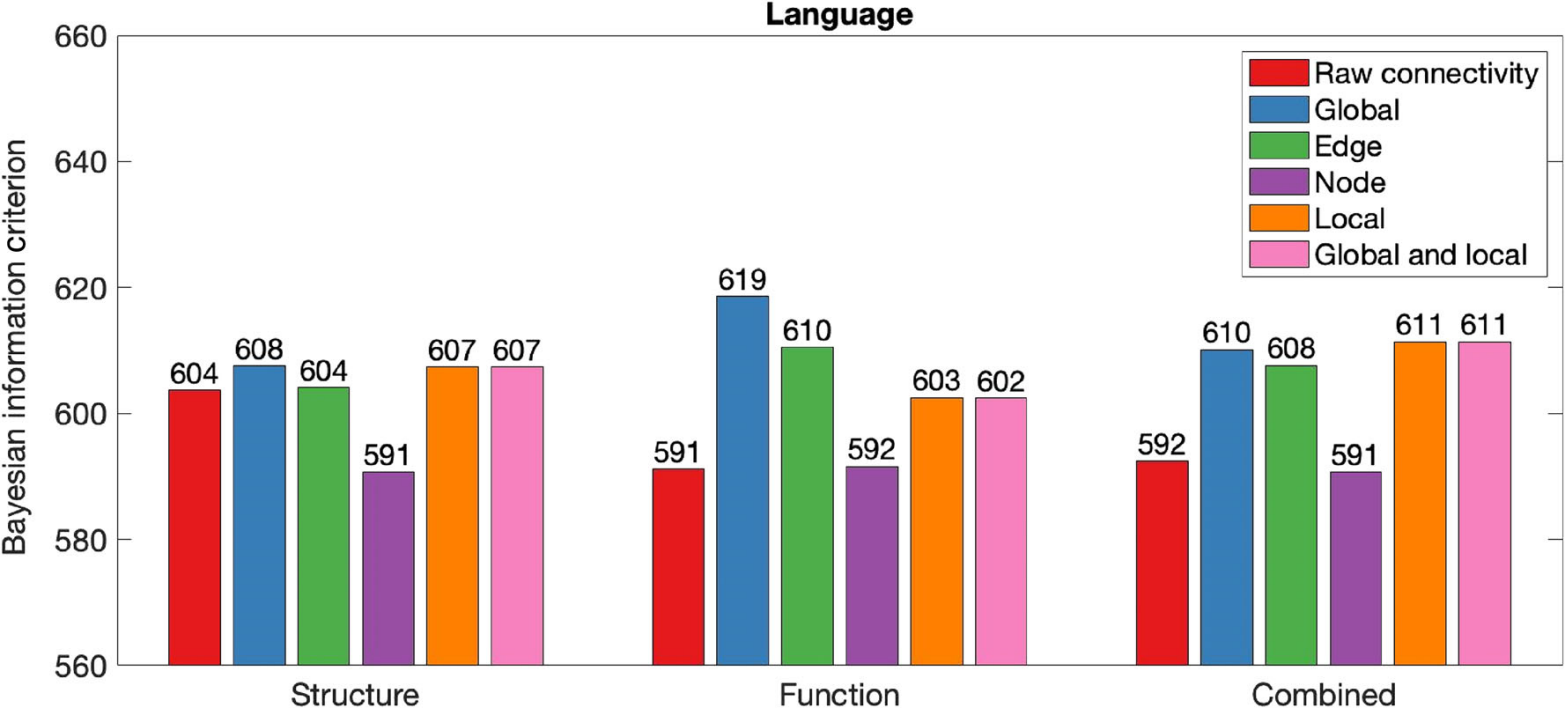
Bayesian information criterion (BIC) model evidence for connectivity and graph theory models of Language. Models with lower BIC values are favoured.

**FIGURE 5:**
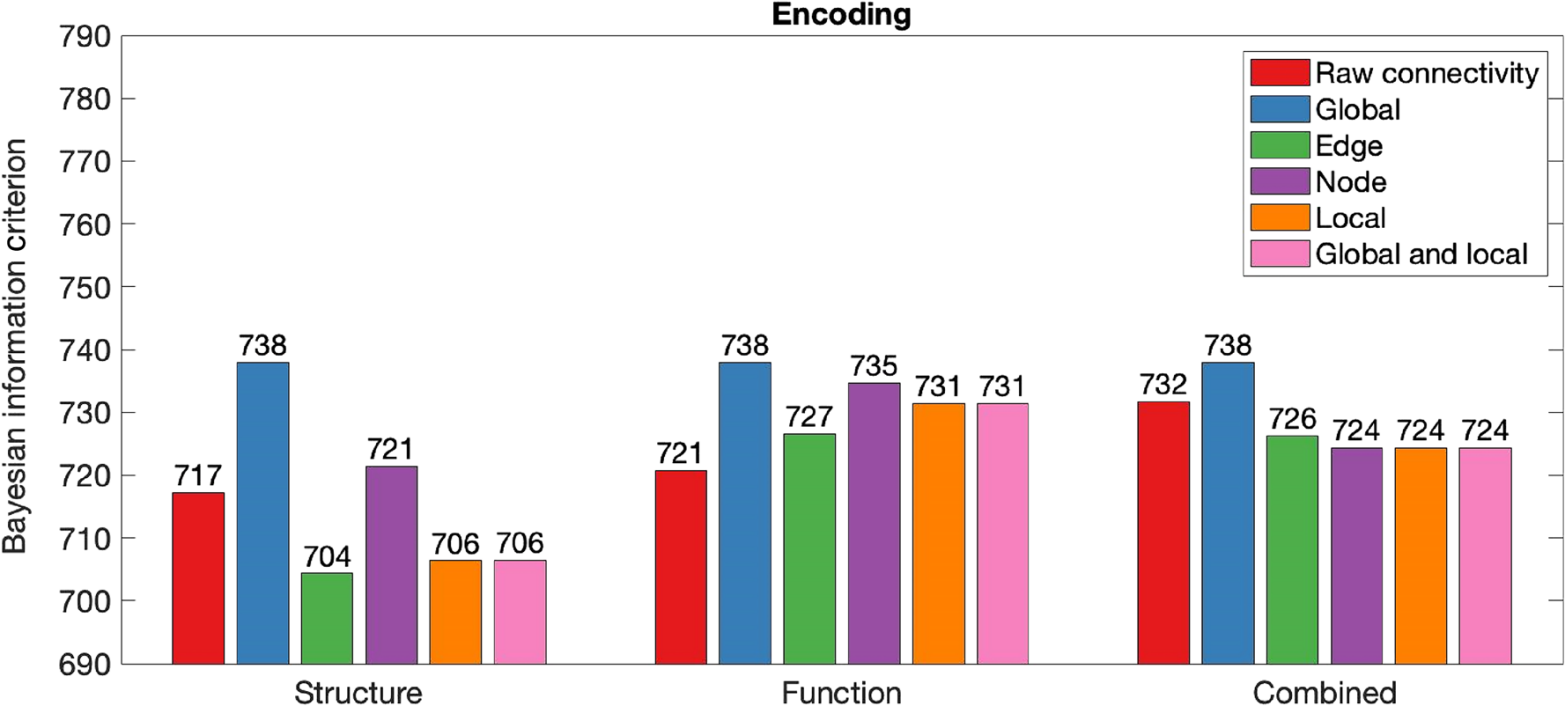
Bayesian information criterion (BIC) model evidence for connectivity and graph theory models of Encoding. Models with lower BIC values are favoured.

**FIGURE 6:**
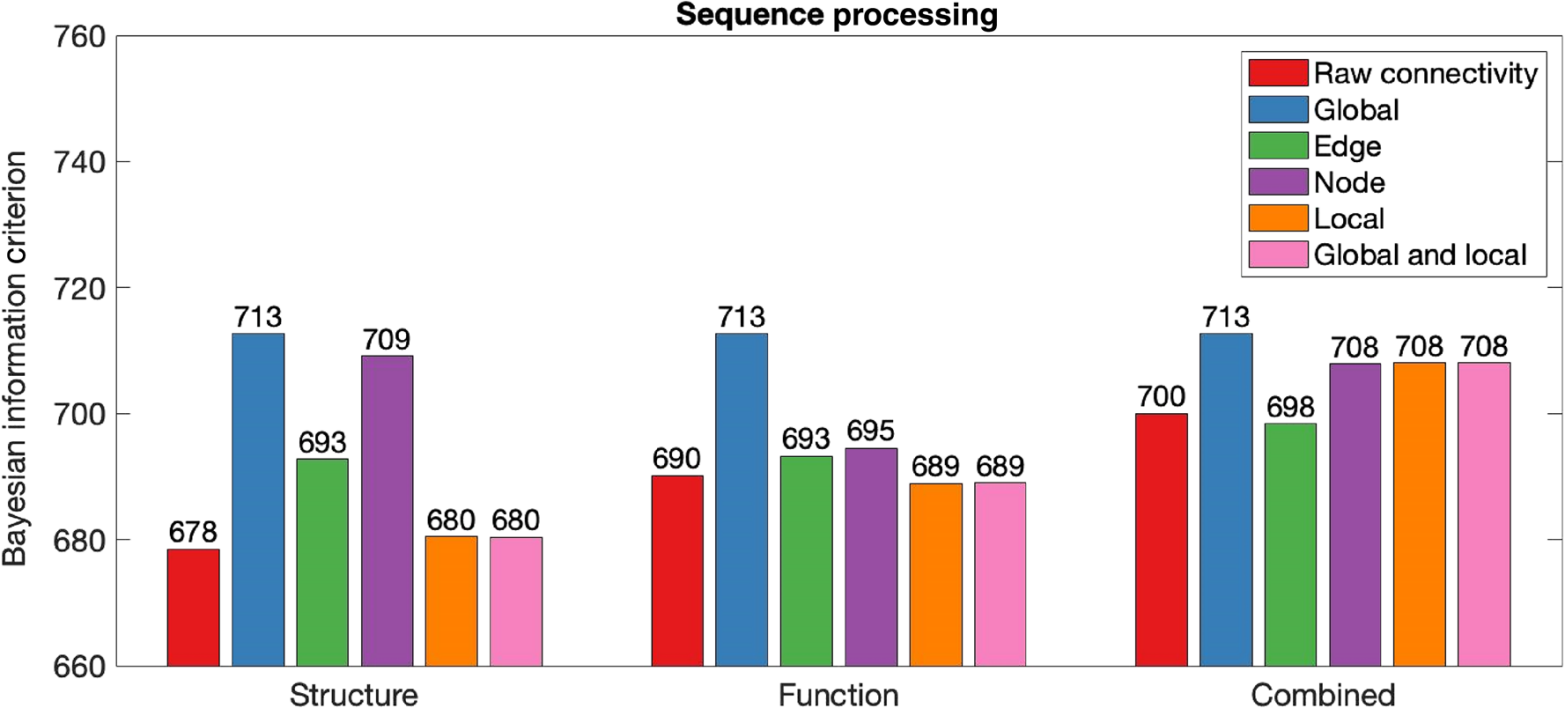
Bayesian information criterion (BIC) model evidence for connectivity and graph theory models of Sequence Processing. Models with lower BIC values are favoured.

Table 2 summarises in‐sample results for models composed with graph theory measures relative to connectivity. Figures 2, 3, 4, 5, 6 illustrate the absolute BIC model evidence values in each cognitive domain.

**TABLE 2:** A summary of in‐sample predictive skill of graph theory models relative to raw connectivity models.

	**Executive function**				
**Modality**	**Global**	**Edge**	**Node**	**Local**	**Global and local**
Structural	12	**−18**	**−2**	**−15**	**−15**
Functional	19	0	17	9	9
Combined	22	1	8	**−5**	**−5**
	**Self‐regulation**				
	**Global**	**Edge**	**Node**	**Local**	**Global and local**
Structural	17	**−7**	15	**−4**	**−4**
Functional	16	**−5**	**−18**	**−15**	**−15**
Combined	15	2	8	11	11
	**Language**				
	**Global**	**Edge**	**Node**	**Local**	**Global and local**
Structural	4	1	**−13**	4	4
Functional	27	19	0	11	11
Combined	18	15	**−2**	19	19
	**Encoding**				
	**Global**	**Edge**	**Node**	**Local**	**Global and local**
Structural	21	**−13**	4	**−11**	**−11**
Functional	17	6	14	11	11
Combined	6	**−5**	**−7**	**−7**	**−7**
	**Sequence Processing**				
	**Global**	**Edge**	**Node**	**Local**	**Global and local**
Structural	34	14	31	2	2
Functional	23	3	4	**−1**	**−1**
Combined	13	**−2**	8	8	8

*Note*: Table values reflect BIC difference. Bold font has been used to indicate models that perform better when graph theory measures are used than when raw connectivity is used.

Abbreviation: BIC, Bayesian information criterion.

When Executive Function was modelled, global graph theory measures proved unable to model cognition across structure, function and their combination. Comparison of BIC values demonstrated moderate evidence that Executive Function was better explained by node graph theory measures of structural network organisation than structural connectivity. Further, there was a strong preference for models composed of edge, local and global and local graph theory measures in the structural modality.

When the functional network was considered, only edge graph theory measures were approximately as effective as raw connectivity in modelling of Executive Function. There was strong evidence favouring the raw connectivity model above all other graph theory measures. When structural and functional information was combined in a single model, joint consideration of local and local and global graph theory measures improved model performance relative to use of combined raw connectivity.

When Self‐regulation was modelled, global graph theory measures proved unable to model cognition across structure, function and their combination. For the structural network, node graph theory measures proved poorer at modelling of Self‐regulation than raw connectivity. However, node, local, and local and global graph theory measures of the structural network were more effective at modelling of Self‐regulation than raw connectivity. For the functional network, node, edge, local, and global and local graph theory measures and were more effective than raw connectivity in modelling of Self‐regulation. When combined structural and functional information was considered, the combined raw connectivity model was preferred above all graph theory measure models.

Model evidence demonstrated that global, edge, local, and local and global graph theory measures were less effective at modelling of Language than raw connectivity. However, node graph theory measures of the structural network have outperformed raw connectivity models. In functional and combined networks, node graph theory measures performed approximately as well as raw connectivity.

Global graph theory measures were not effective at modelling of Encoding. Node graph theory measures of structural networks were less effective at modelling of Encoding than raw connectivity. However, models produced with edge, local, and global and local graph theory of the structural network were favoured above raw connectivity models. Model evidence demonstrated that functional raw connectivity outperformed all graph theory measures in modelling of Encoding. Model evidence favoured models constructed with node, edge, local, and global and local graph theory measures relative to the model constructed with combined raw connectivity.

Finally, Sequence Processing was not effectively modelled by global graph theory measures. All graph theory measures of the structural network were less effective than raw connectivity in modelling of Sequence Processing, although the difference between raw connectivity models and models composed of local and local and global graph theory measures was not significant. The functional raw connectivity model was preferred above the model produced with node and edge graph theory measures. The model produced with local graph theory measures of the functional network preferred above the raw connectivity model. When combined structural‐functional information was considered, the model constructed with edge graph theory measures was somewhat preferable to the raw connectivity model.

#### 3.1.1 Cross‐validation based model comparison

**FIGURE 7:**
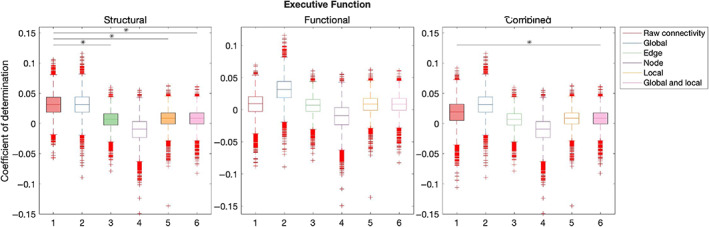
Results of BBC‐CV of Executive Function, as measured by the coefficient of determination. The solid lines show the median scores, the boxes show the interquartile range (IQR), and ticks outside of whiskers indicate outlier scores across all bootstrap samples. Filled boxes illustrate greater than chance prediction and unfilled boxes illustrate not greater than chance prediction. The asterisks indicate significant differences (*p* < .001) between connectivity‐based and graph theory‐based model coefficients of determination observed for models that perform significantly better than chance.

**FIGURE 8:**
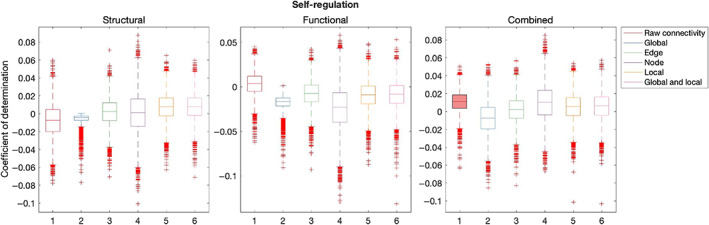
Results of BBC‐CV of Self‐regulation, as measured by the coefficient of determination. The solid lines show the median scores, the boxes show the interquartile range (IQR), and ticks outside of whiskers indicate outlier scores across all bootstrap samples. Filled boxes illustrate greater than chance prediction and unfilled boxes illustrate not greater than chance prediction. The asterisks indicate significant differences (*p* < .001) between connectivity‐based and graph theory‐based model coefficients of determination observed for models that perform significantly better than chance.

**FIGURE 9:**
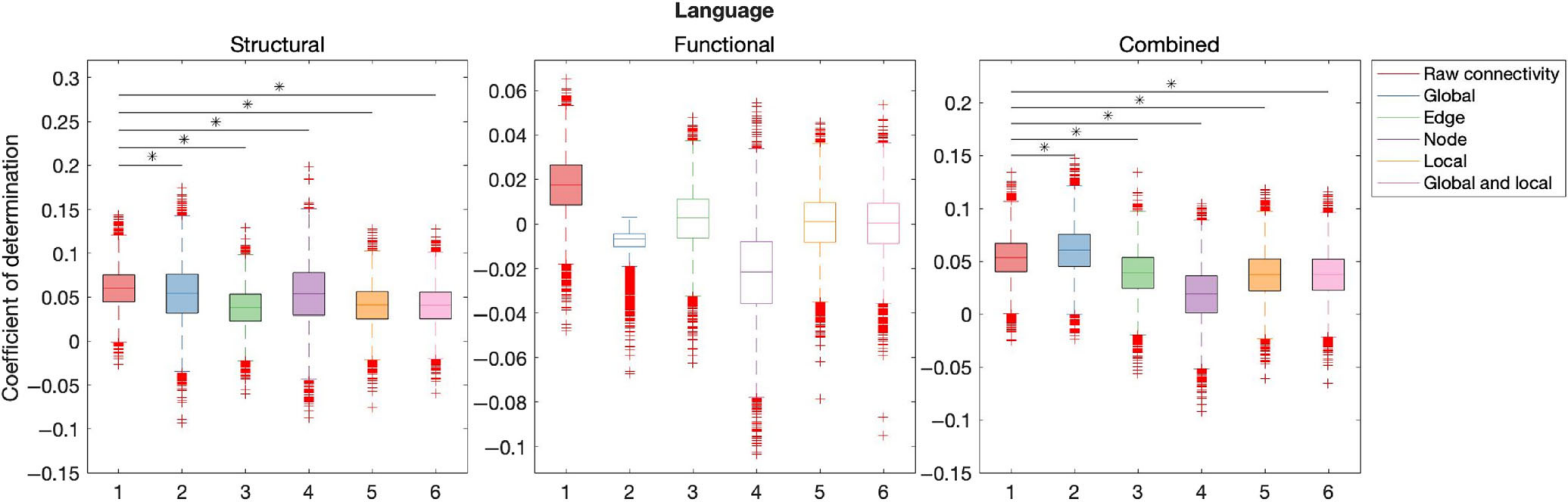
Results of BBC‐CV of Language, as measured by the coefficient of determination. The solid lines show the median scores, the boxes show the interquartile range (IQR), and ticks outside of whiskers indicate outlier scores across all bootstrap samples. Filled boxes illustrate greater than chance prediction and unfilled boxes illustrate not greater than chance prediction. The asterisks indicate significant differences (*p* < .001) between connectivity‐based and graph theory‐based model coefficients of determination observed for models that perform significantly better than chance.

**FIGURE 10:**
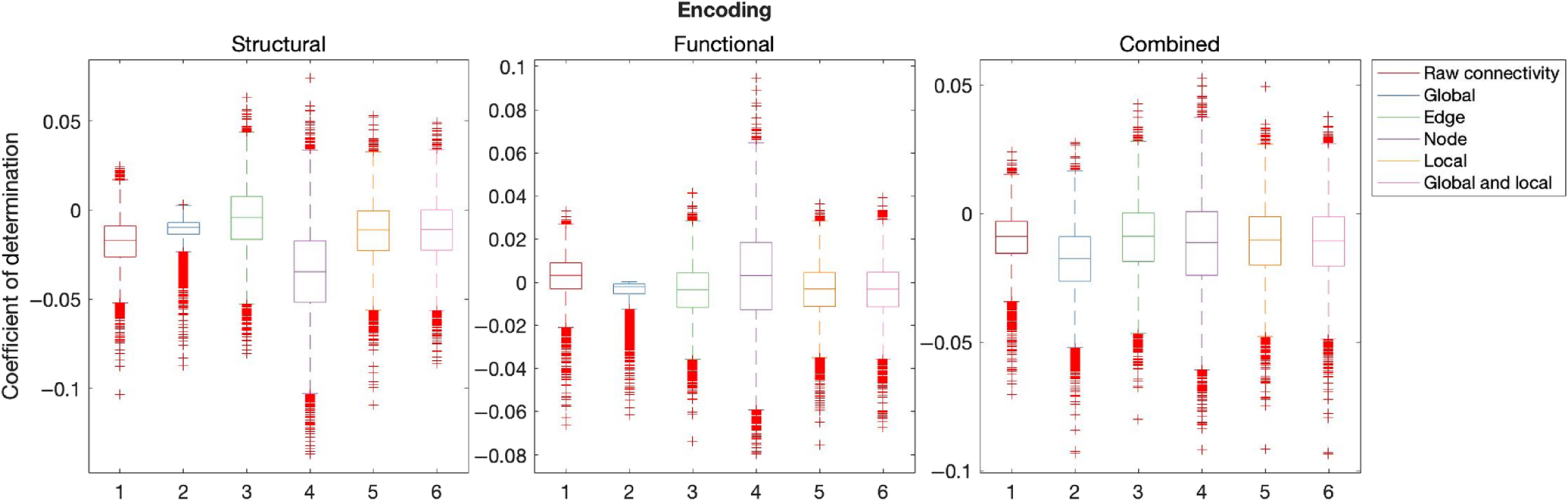
Results of BBC‐CV of Encoding, as measured by the coefficient of determination. The solid lines show the median scores, the boxes show the interquartile range (IQR), and ticks outside of whiskers indicate outlier scores across all bootstrap samples. Filled boxes illustrate greater than chance prediction and unfilled boxes illustrate not greater than chance prediction. The asterisks indicate significant differences (*p* < .001) between connectivity‐based and graph theory‐based model coefficients of determination observed for models that perform significantly better than chance.

**FIGURE 11:**
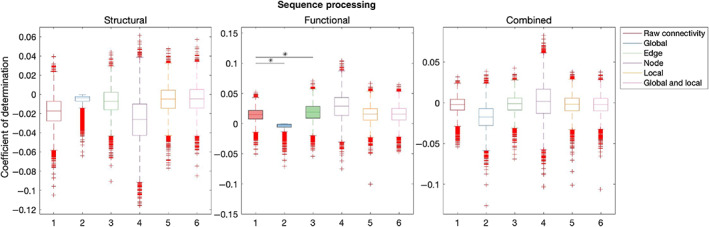
Results of BBC‐CV of Sequence Processing, as measured by the coefficient of determination. The solid lines show the median scores, the boxes show the interquartile range (IQR), and ticks outside of whiskers indicate outlier scores across all bootstrap samples. Filled boxes illustrate greater than chance prediction and unfilled boxes illustrate not greater than chance prediction. The asterisks indicate significant differences (*p* < .001) between connectivity‐based and graph theory‐based model coefficients of determination observed for models that perform significantly better than chance.

Table 3 summarises out‐of‐sample results for models composed with graph theory measures relative to connectivity. Figures 7, 8, 9, 10, 11 illustrate the results of the BBC‐CV procedure, as measured by the coefficient of determination. Filled boxes illustrate greater‐than‐chance prediction skill. Only results for models that predict greater than chance will be considered further and the analysis will compare the performance of connectivity‐based and graph theory‐based models.

**TABLE 3:** A summary of out‐of‐sample predictive skills of graph theory models relative to raw connectivity models.

Modality	Global	Edge	Node	Local	Global and local
Structural	–	85.86		82.28	82.02
Functional	–	–	–	–	–
Combined	–	–	–	–	40.26
	Global	Edge	Node	Local	Global and local
Structural	–	–	–	–	–
Functional	–	–	–	–	–
Combined	–	–	–	–	–
	Language				
	Global	Edge	Node	Local	Global and local
Structural	14.45	62.8	14.95	55.45	56.27
Functional	–	–	–	–	–
Combined	**−22.04**	46.07	–	51.19	50.97
	Encoding				
	Global	Edge	Node	Local	Global and local
Structural	–	–	–	–	–
Functional	–	–	–	–	–
Combined	–	–	–	–	–
	Sequence processing				
	Global	Edge	Node	Local	Global and local
Structural	–	–	–	–	–
Functional	106.84	**−22.13**	–	–	–
Combined	–	–	–	–	–

*Note*: Table values reflect Wilcoxon rank sum values *Z* score (all *p*‐values are <.001). Bold font has been used to indicate models that perform better when graph theory measures are used than when raw connectivity is used.

In structural networks, raw connectivity models (mean = 0.03, SD = 0.02) explained more variation in the Executive Function of the validation sample than the edge (mean = 0.01, SD = 0.02) (*Z* = 85.86, *p* < .001), local (mean = 0.01, SD = 0.01) (*Z* = 82.28, *p* < .001), and local and global (mean = 0.01, SD = 0.01) (*Z* = 82.02, *p* < .001) graph theory models. Models combining structural and functional information explained significantly more variation using raw connectivity (mean = 0.02, SD = 0.02) than global and local graph theory measures (mean = 0.01, SD = 0.01) (*Z* = 40.26, *p* < .001).

Only the combined raw connectivity model of Self‐regulation performed above chance (mean = 0.01, SD = 0.01).

In structural modality, raw connectivity models (mean = 0.06, SD = 0.02) explained significantly more variation in the Language of the validation sample than global (mean = 0.05, SD = 0.03) (*Z* = 14.45, *p* < .001), edge (mean = 0.3, SD = 0.02) (*Z* = 62.8, *p* < .001), node (mean = 0.05, SD = 0.04) (*Z* = 14.95, *p* < .001), local (mean = 0.05, SD = 0.02) (*Z* = 55.45, *p* < .001), and local and global (mean = 0.04, SD = 0.02) (*Z* = 56.27, *p* < .001) models. In functional modality, only raw connectivity models explained more variation in Language than chance (mean = 0.02, SD = 0.01). When combined structural‐functional models were considered, raw connectivity (mean = 0.05, SD = 0.02) explained significantly more variation in Language than the edge (mean = 0.04, SD = 0.02) (*Z* = 46.07, *p* < .001), node (mean = 0.02, SD = 0.03) (*Z* = 87.11, *p* < .001), local (mean = 0.04, SD = 0.02) (*Z* = 51.19, *p* < .001), local and global (mean = 0.04, SD = 0.02) (*Z* = 50.97, *p* < .001) graph theory measures but not the global graph theory measures (mean = 0.06, SD = 0.02) (*Z* = −22.04, *p* < .001).

No model of Encoding could produce results that were more generalizable to unseen samples than chance.

Finally, when Sequence Processing was considered, only the functional network could produce generalizable models of cognition. Raw connectivity models (mean = 0.01, SD = 0.01) explained more variation in Sequence Processing than global graph theory measures (mean = −0.01, SD = 0.01) (*Z* = 106.84, *p* < .001). However, functional edge graph theory measures explained more variation than raw connectivity (mean = 0.02, SD = 0.02) (*Z* = −22.13, *p* < .001).

## 4 DISCUSSION

Graph theory has been previously used to quantify network organization and relate it to cognitive function but little work has been done to assess its value in predictive models (Farahani et al., 2019). In this work, we constructed predictive regression models of cognitive function with graph theory measures. We compared the predictive performance of graph theory‐based models to models constructed with raw connectivity. To achieve this goal, a series of models were constructed with the SWR‐PCR approach. Their in‐sample performance was assessed by comparing BIC model evidence and their generalizability was assessed by comparing their predictive performance of unseen datasets. Local and global graph theory measures could be used to predict cognitive performance in a healthy adult sample. However, they could not consistently outperform raw connectivity at the quality of in‐sample and out‐of‐sample predictions.

In this work, we explored modelling of five different cognitive domains using a collection of 19 graph theory measures from structural and functional connectivity. Global graph theory models have only succeeded at predicting in‐sample Language abilities. When out‐of‐sample predictive ability was considered, global graph theory measures were able to predict Language abilities for structural and combined models, and Sequence Processing abilities for functional models. This demonstrates that in this healthy population global network characteristics predict linguistic abilities and predict Sequence Processing but their relationship to other cognitive domains is weak. This finding was largely contrary to our expectations. Highly complex and abstract tasks have been demonstrated to engage large parts of the distributed network. Consequently, we expected that understanding the global organization of the brain will benefit the explanation of high‐order cognitive domains like Executive Function and Self‐regulation. Previous work has successfully demonstrated that global graph theory can effectively be used in prediction of diagnosis and cognitive function of epilepsy, attention deficit hyperactivity disorder and dementia (Colby et al., 2012; Hojjati et al., 2017; Sethi et al., 2019). The difference between previous work and present findings suggests that while network disruption may be observed for atypical populations and allow successful classification, it is not clear if this network organization maps directly onto various cognitive domains observed in a healthy population. Consequently, we advise caution during the interpretation of graph theory measure models across populations.

We have also found that various combinations of local graph theory measures have succeeded at modelling of all cognitive domains to varying degrees. Graph theory measures occasionally outperformed raw connectivity at predicting cognition. For example, Executive Function was more effectively explained by local graph theoretic measures of the structural network than SC. However, the graph theory results were erratic, as no specific collection of graph theory measures could consistently explain cognition across domains or modalities more effectively than others. This means that on some occasions information about node embedding within the network was more effective at explaining cognitive performance, but on other occasions information about edge embedding within the network was more effective. While it is not necessarily the case that one would expect complete consistency across cognitive domains, there was no discernable pattern that would allow formulation of theories of cognition and its relation to structure and function.

In summary, we did not find a consistent benefit to the explanation and prediction of domains of healthy and typical cognitive domains from the use of graph theory relative to the use of connectivity values. This demonstrates that when studying individual differences in cognition, connectivity effectively captures variation in the brain networks related to cognition. Representation of variation with graph theory does not add to explanatory capacity of models of cognition. This finding is likely due to the nature of graph theory—it summarizes information and thus provides an interpretable overview of network organization. This summary would benefit modelling of cognition if it would remove irrelevant information that does not benefit the explanation of cognition. For example, strength does not reflect what pattern of edges contributes to node strength. Consequently, the use of graph theory measures results in a loss of some information about connectivity that is relevant to cognitive domains. Taken together, these results suggest that the interpretation of results of graph theory models must be approached with caution. Graph theory presents a meaningful representation of network organization and information exchange. However, on its own graph theory does not appear to be very effective at modelling of cognition in a healthy population, and this may call into question the extent to which abnormalities in graph theory measures seen in atypical populations are meaningful.

This work has also proposed that there is a fundamental difference between what information is expressed by structural and functional connectivity in that structural connectivity expresses physical connections between pairs of nodes, whereas functional connectivity expresses statistical associations in their activation during rest. We reasoned that graph theory quantifies the organisational properties of networks and thereby it provides a common language for structural and functional information. Consequently, we also expected that graph theory would aid the efficiency of modelling when combined structural‐functional information was considered. However, in combined structural‐functional models, consideration of connectivity values sometimes proved more effective at modelling of cognition than combinations of graph theory measures. When structural and functional information was combined, models constructed with graph theory did not outperform connectivity at explaining and predicting cognitive information. This is a very important finding because it demonstrates that the fact that structural and functional information express different information about the state of the brain does not impede its combination in a common model, and may improve it. This validates the previous endeavours of modelling cognition using standard measures of structural and/or functional brain connectivity (Dhamala et al., 2021; Litwińczuk et al., 2022; Rasero et al., 2021). Furthermore, this finding adds to the previous literature by demonstrating that the advantage of combining structural and functional information in modelling of cognition is due to divergent information expressed by structural and functional connectivity and it appears that this divergence is better captured in connectivity than graph theory.

Several methodological caveats must be considered when assessing the results of the present work. Here, the PCA‐SWR approach was implemented to produce predictive models of cognition. The present work only considered linear models. It remains a possibility that non‐linear associations exist between connectivity and cognition. In addition, previous work has demonstrated that SWR selects different features across samples (Nogueira et al., 2017). It is possible that more consistent findings could be obtained with the introduction of feature selection before model training. Previous research also demonstrates that many predictive methods (e.g., lasso, connectome‐based predictive modelling) tend to produce different beta weights across samples (Tian & Zalesky, 2021). Consequently, implementation of an alternative regression method and obtainment of average beta weights across repetitions of cross‐validation may benefit model generalisability.

Overall, this work has demonstrated that graph theory can be used to model healthy performance across cognitive domains. Yet there was no notable benefit to regression modelling conducted with graph theory measures relative to the use of structural, functional and combined structural‐functional connectivity. Hence while graph theory may represent meaningful information about the state of the system, it did not produce consistent improvements in explanation or predictions across cognitive domains. While graph theory may prove useful to understand the characteristics of the neural network organization in atypical populations, our work brings to question whether such findings map meaningfully onto cognitive performance of healthy adults.

## FUNDING INFORMATION

Biotechnology and Biological Sciences Research Council, UK (grant reference: BB/M011208/1). Data were provided by the Human Connectome Project, WU‐Minn Consortium (Principal Investigators: David Van Essen and Kamil Ugurbil; 1U54MH091657) funded by the 16 NIH Institutes and Centers that support the NIH Blueprint for Neuroscience Research; and by the McDonnell Center for Systems Neuroscience at Washington University.

## CONFLICT OF INTEREST STATEMENT

The authors declare no conflicts of interest.

## Supporting information


**Appendix S1:** Supporting information.

## Data Availability

Data is openly available as part of the WU‐Minn HCP 1200 Subjects Data Release of HCP Young Adult study, part of the Human Connectome Project (https://www.humanconnectome.org/study/hcp-young-adult/). Codes for data analysis are available at https://github.com/MCLit/GT-PCA-SWR/.
