# Supplementary material for: Using graph theory as a common language to combine neural structure and function in models of healthy cognitive performance
Source: Hum Brain Mapp. 2023 Mar 7;44(8):3007–22. doi: 10.1002/hbm.26258 (PMC10171528; doi:10.1002/hbm.26258)
Supplement: Supplementary file 1 — Appendix S1: Supporting information. [file HBM-44-3007-s001.docx]

**Supplementary Material 1**

Supplementary Figures 1-5 illustrate Bayesian Information Criterion (BIC) obtained from SWR-PCR when regression models were separately produced for each local graph theory measure.


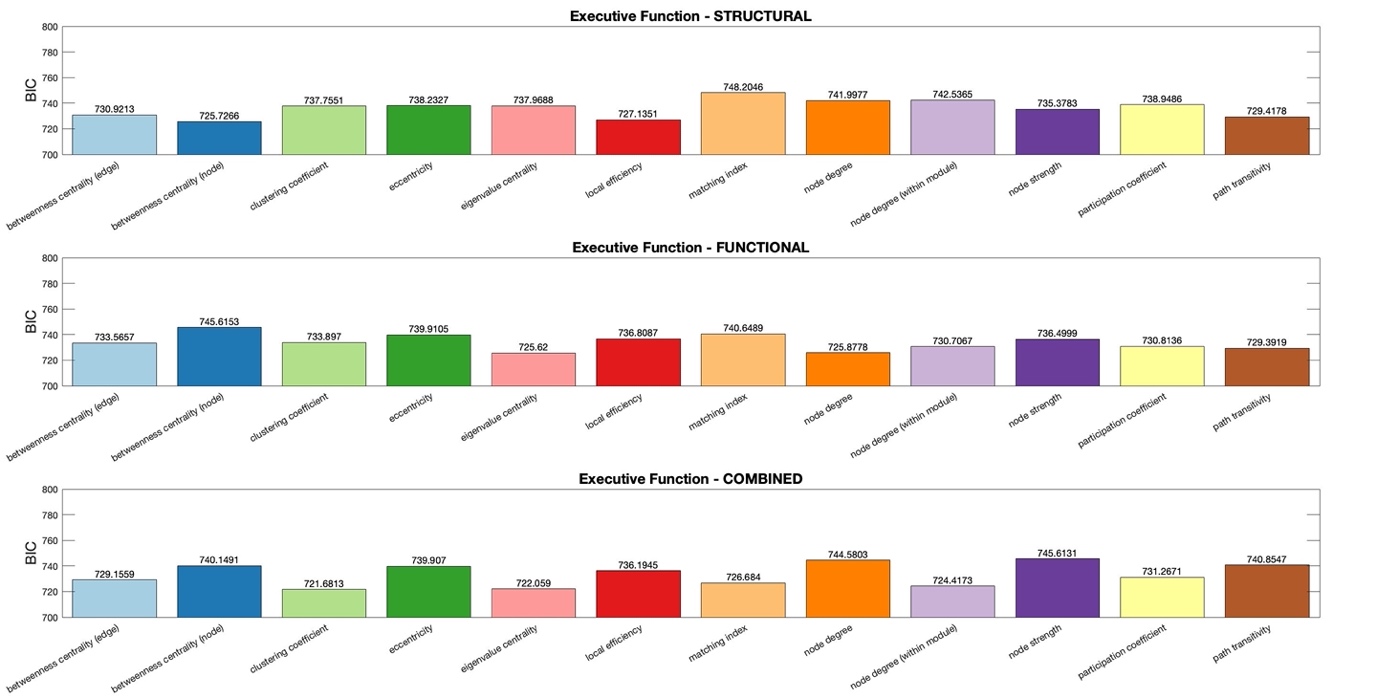


Supplementary Figure 1 BIC model evidence for graph theory models of Executive Function. Models with lower BIC values are favoured.


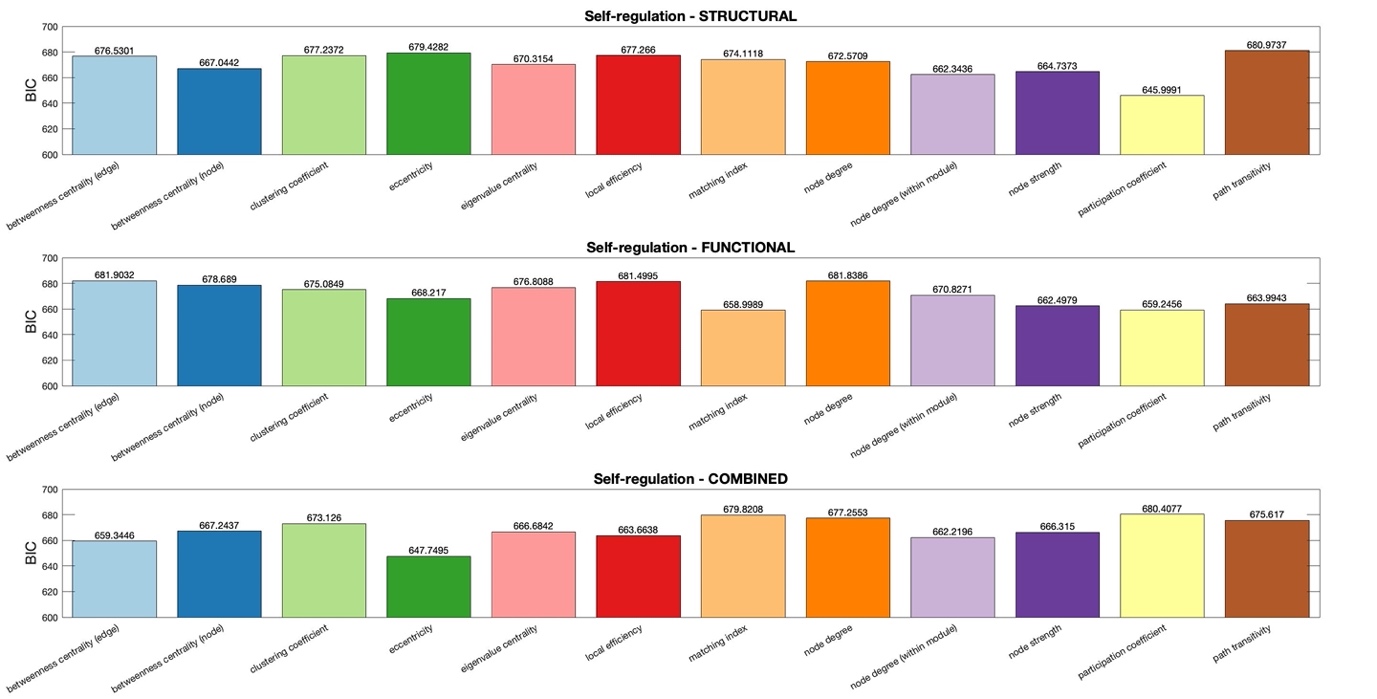


Supplementary Figure 2 BIC model evidence for graph theory models of Self-regulation. Models with lower BIC values are favoured.


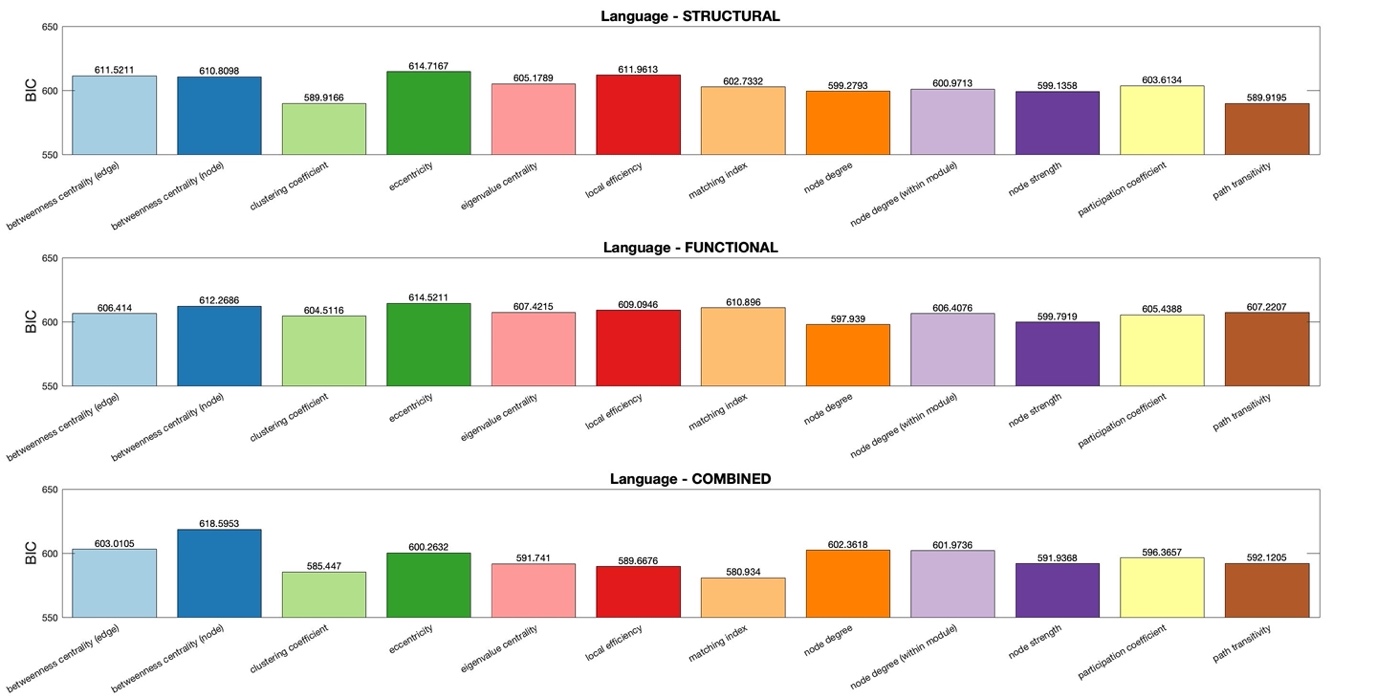


Supplementary Figure 3 BIC model evidence for graph theory models of Language. Models with lower BIC values are favoured.


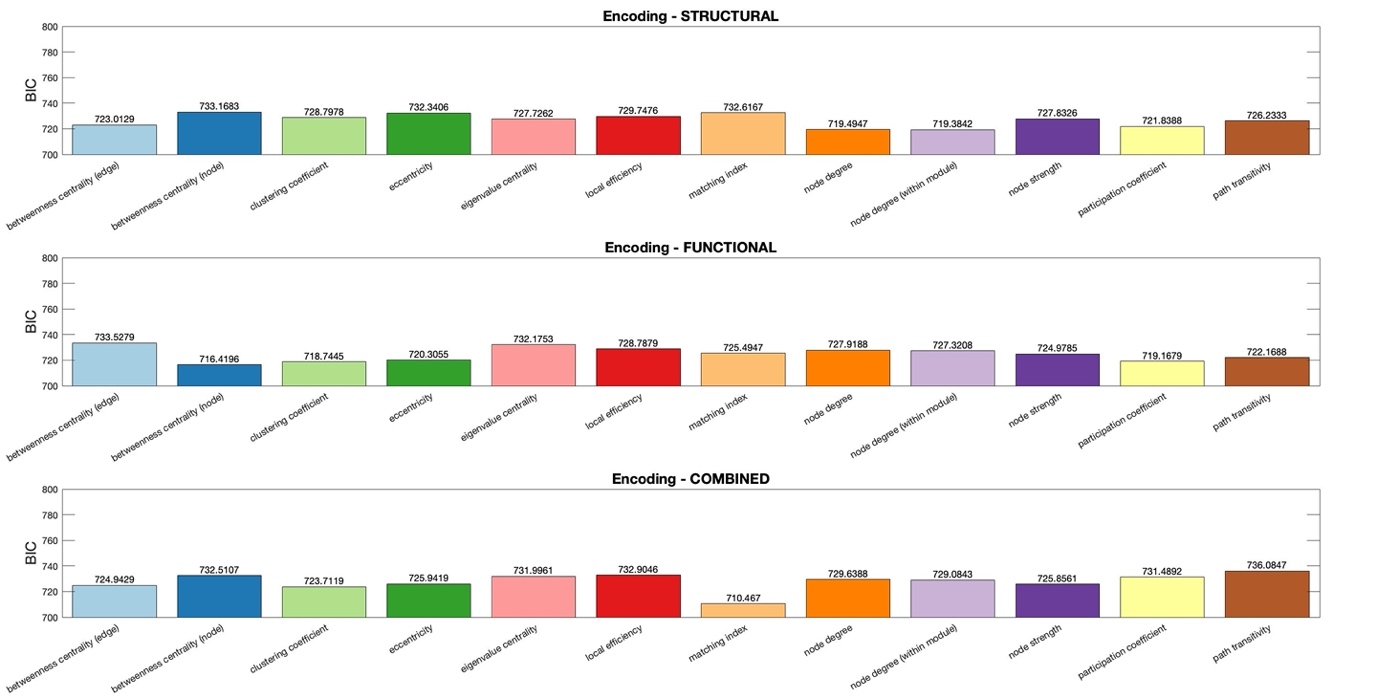


Supplementary Figure 4 BIC model evidence for graph theory models of Encoding. Models with lower BIC values are favoured.


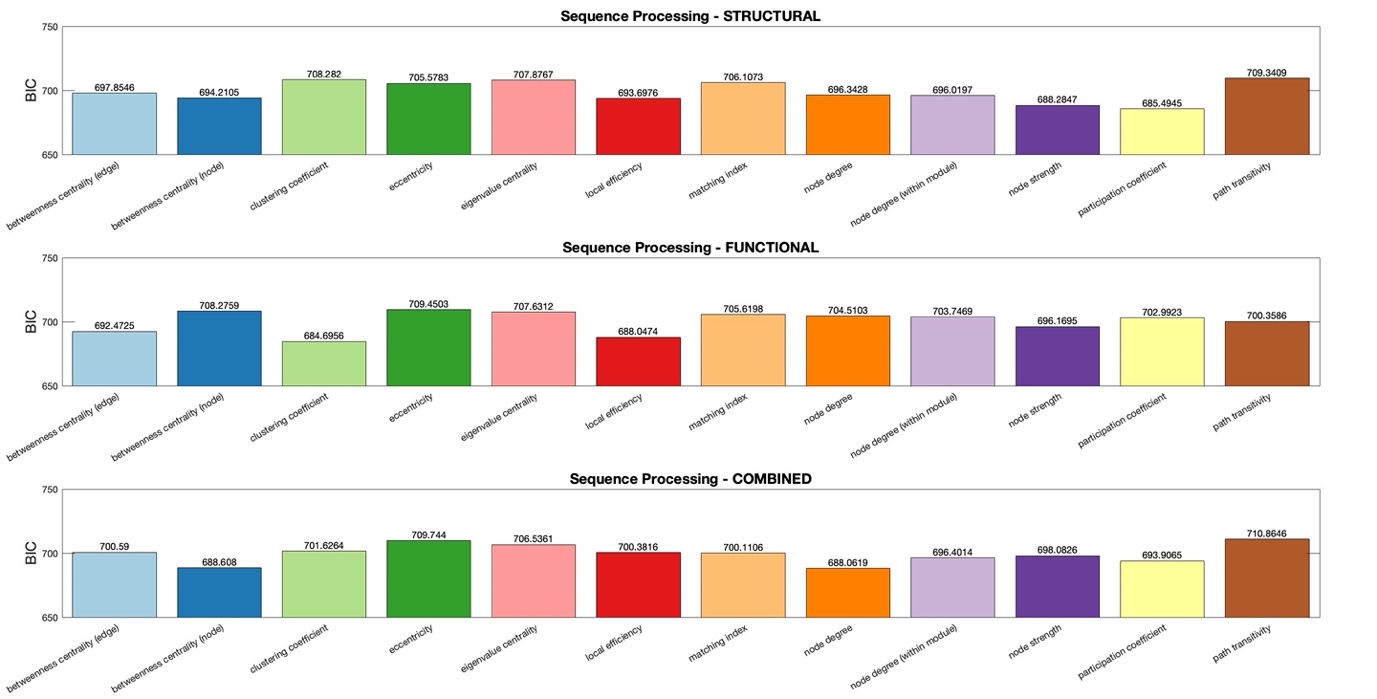


Supplementary Figure 5 BIC model evidence for graph theory models of Sequence Processing. Models with lower BIC values are favoured.

Supplementary Figures 6-10 illustrate model predictive skill obtained during cross-validation with SWR-PCR when regression models were separately produced for each local graph theory measure.


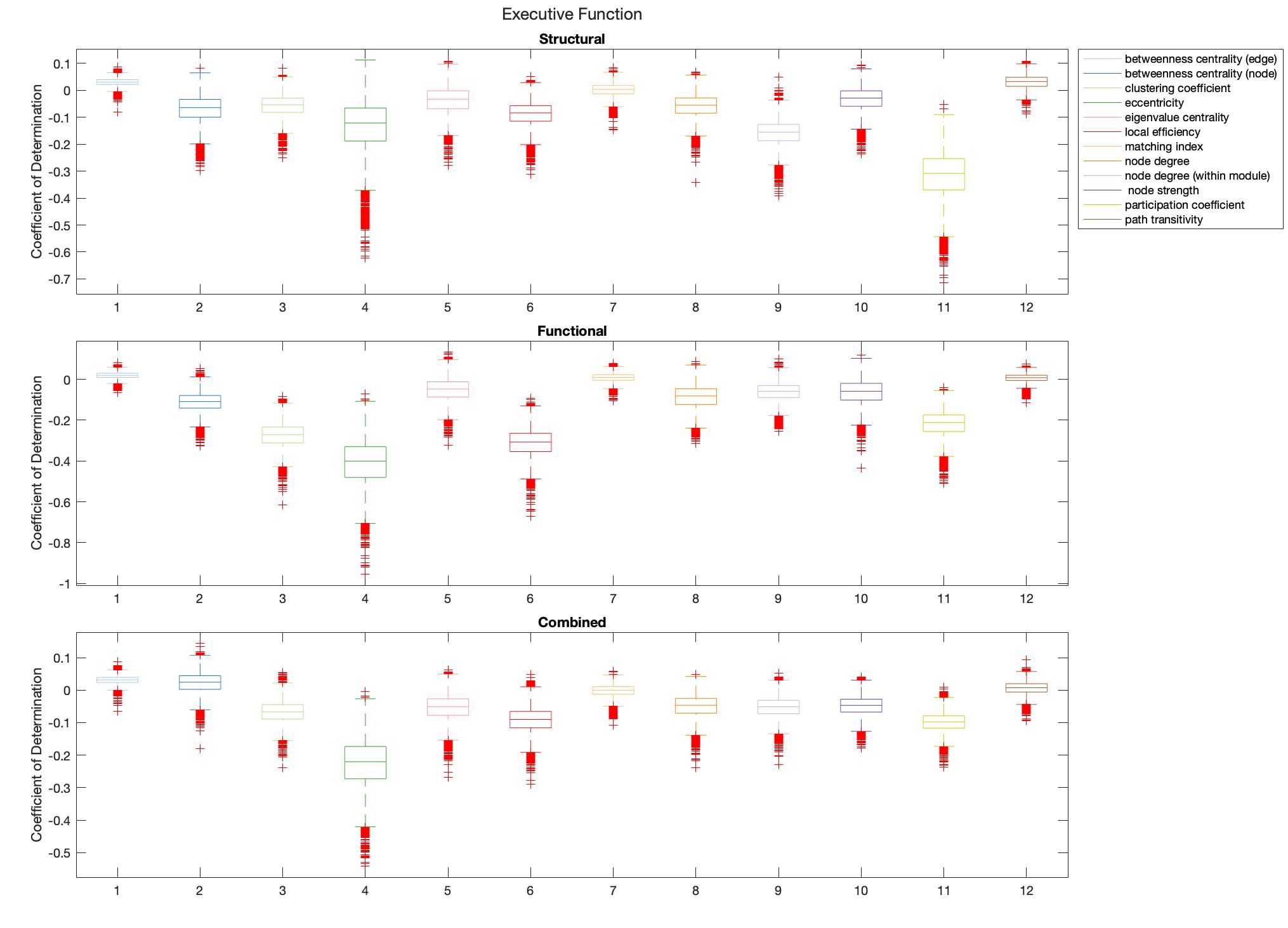


Supplementary Figure 6 Results of BBC-CV of Executive Function, as measured by coefficient of determination. The solid lines show the median scores, the boxes show the interquartile range (IQR), and ticks outside of whiskers indicate outlier scores across all bootstrap samples.


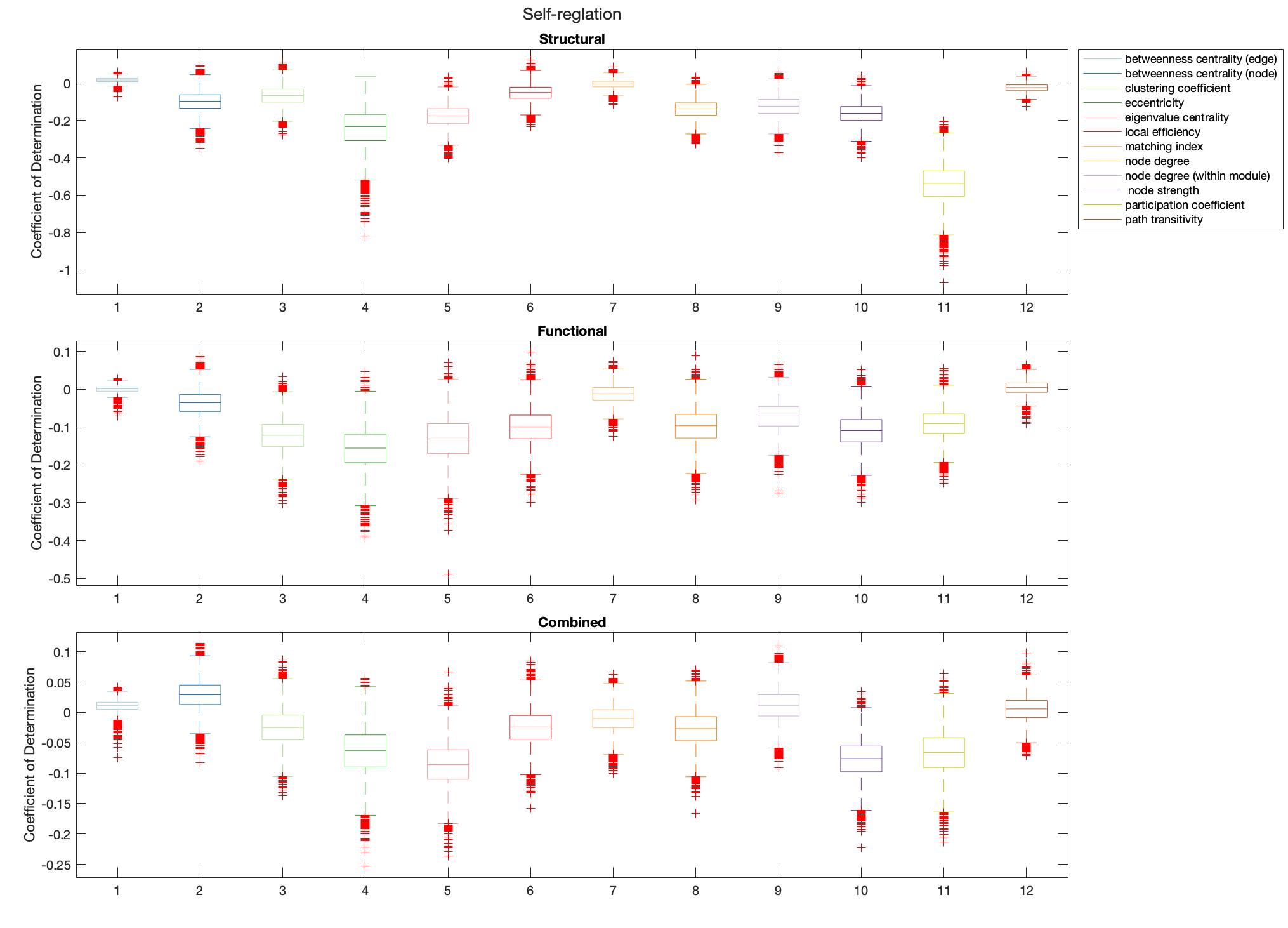


Supplementary Figure 7 Results of BBC-CV of Self-regulation, as measured by coefficient of determination. The solid lines show the median scores, the boxes show the interquartile range (IQR), and ticks outside of whiskers indicate outlier scores across all bootstrap samples.


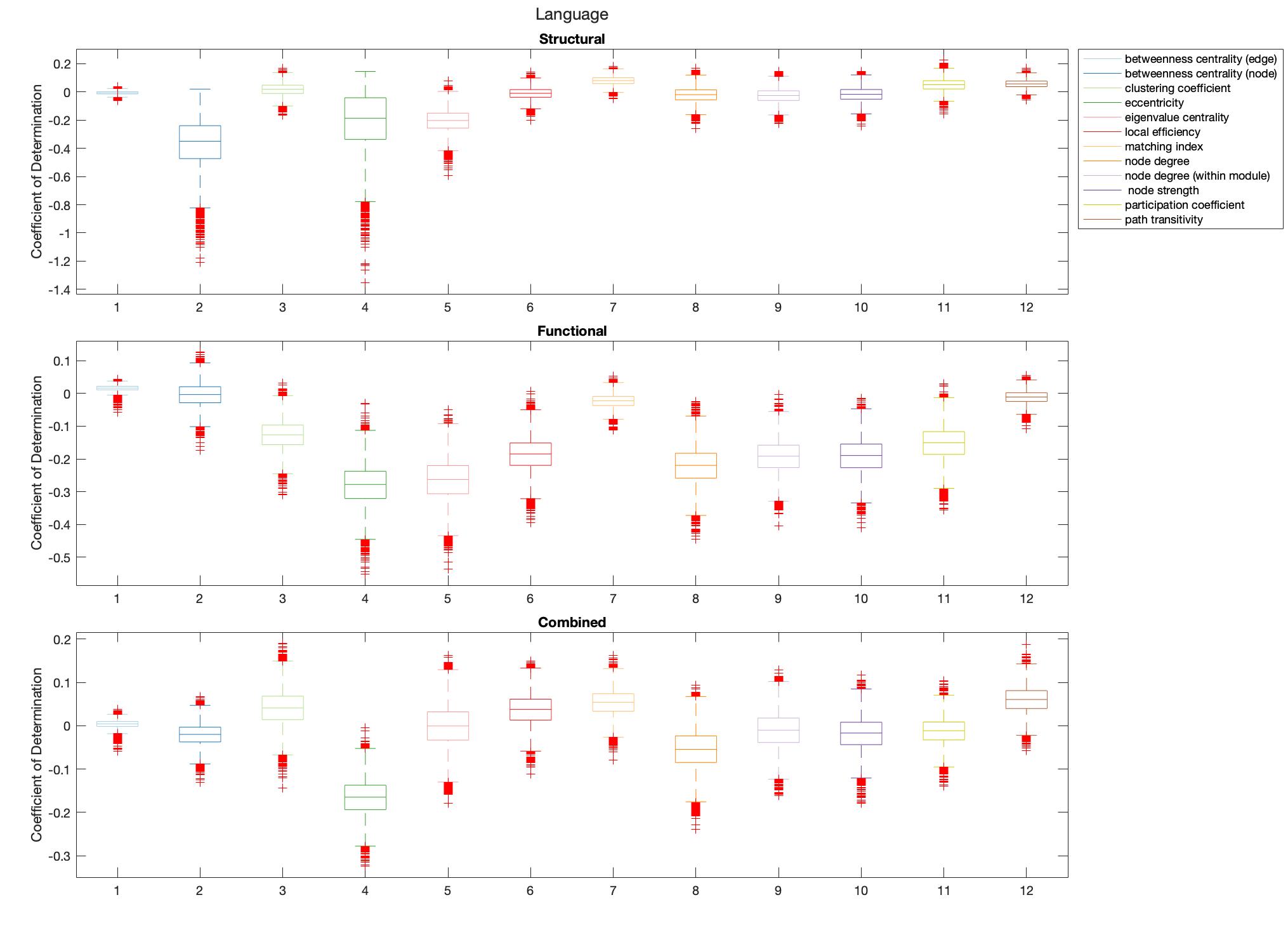


Supplementary Figure 8 Results of BBC-CV of Language, as measured by coefficient of determination. The solid lines show the median scores, the boxes show the interquartile range (IQR), and ticks outside of whiskers indicate outlier scores across all bootstrap samples.


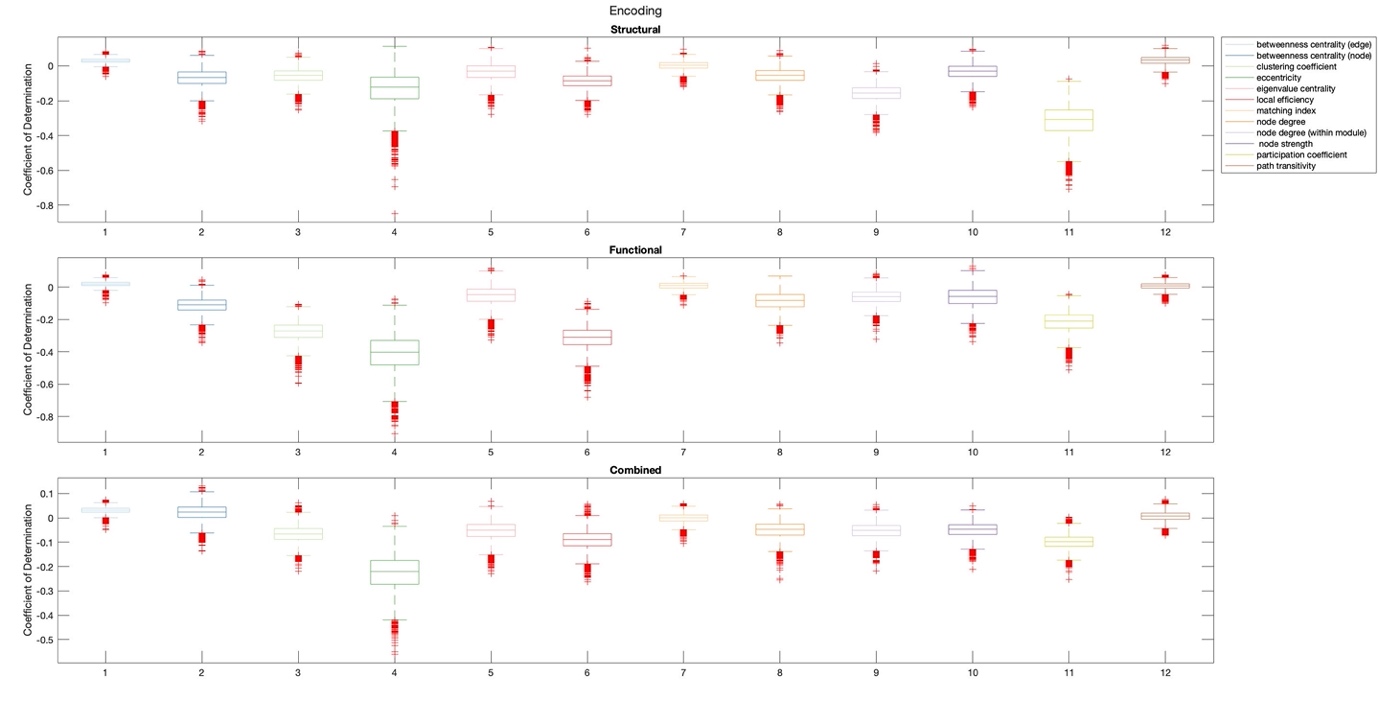


Supplementary Figure 9 Results of BBC-CV of Encoding, as measured by coefficient of determination. The solid lines show the median scores, the boxes show the interquartile range (IQR), and ticks outside of whiskers indicate outlier scores across all bootstrap samples.


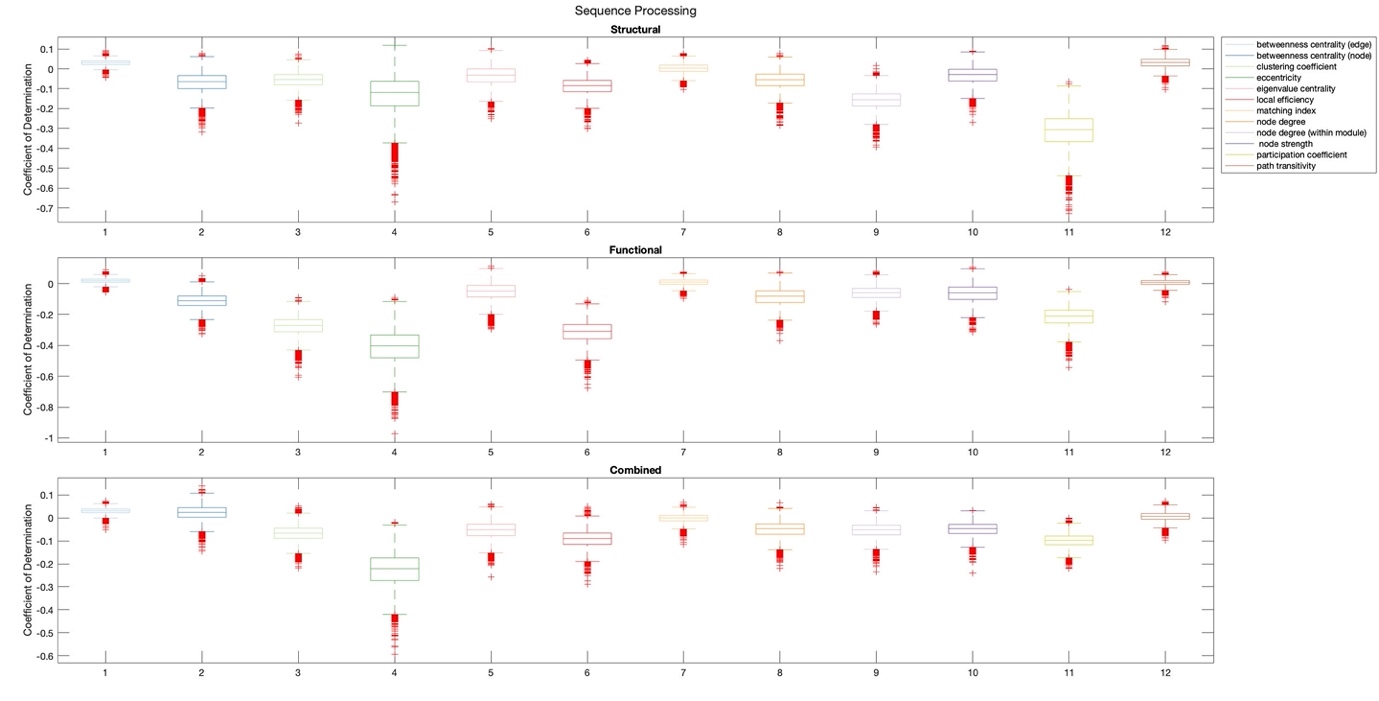


Supplementary Figure 10 Results of BBC-CV of Sequence Processing, as measured by coefficient of determination. The solid lines show the median scores, the boxes show the interquartile range (IQR), and ticks outside of whiskers indicate outlier scores across all bootstrap samples.

**Supplementary Material 2**

Supplementary Figures 6-10 illustrate Coefficients of determination for models obtained in the main manuscript with SWR-PCR when regression models were separately produced for connectivity values and various combinations of graph theory measures.


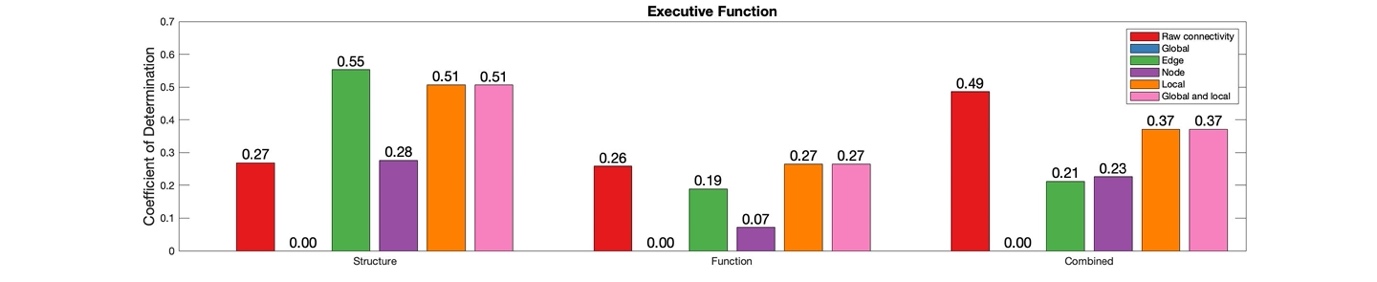


Supplementary Figure 11 Ordinary R-Squared for models of Executive Function.


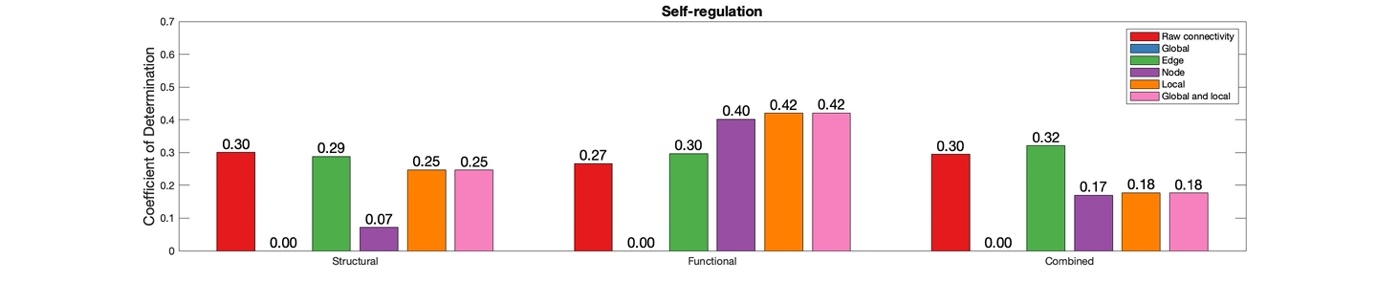


Supplementary Figure 12 Ordinary R-Squared for models of Self-regulation.


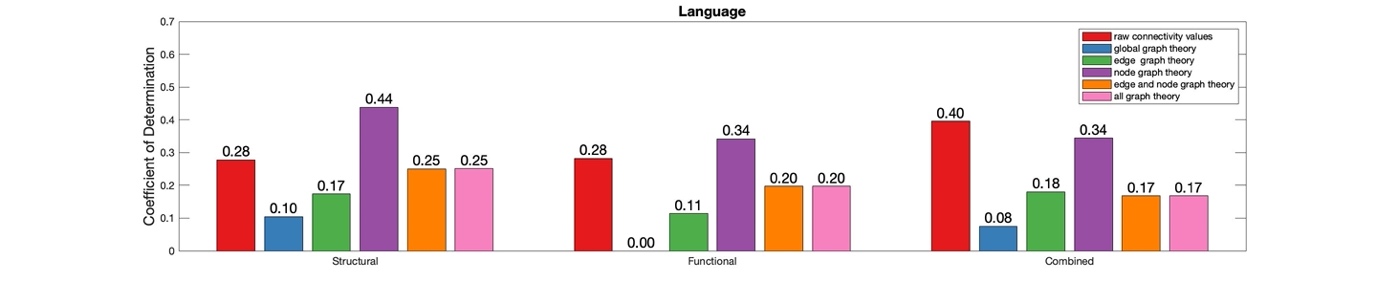


Supplementary Figure 13 Ordinary R-Squared for models of Language.


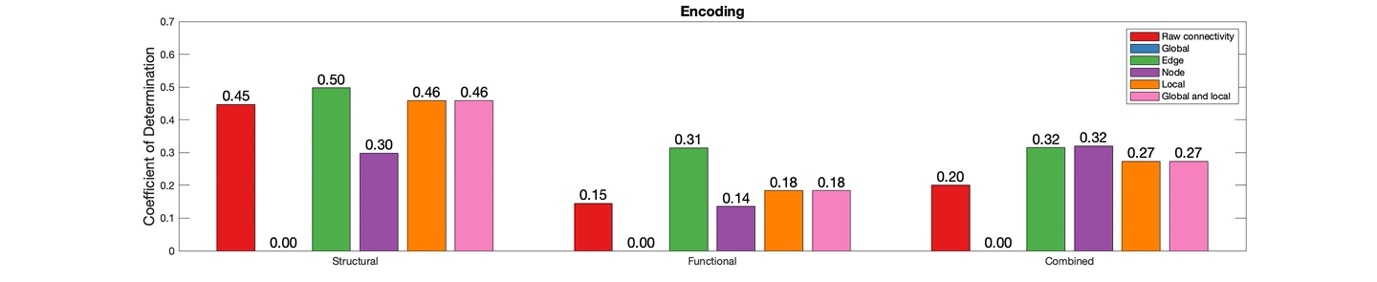


Supplementary Figure 14 Ordinary R-Squared for models of Encoding.


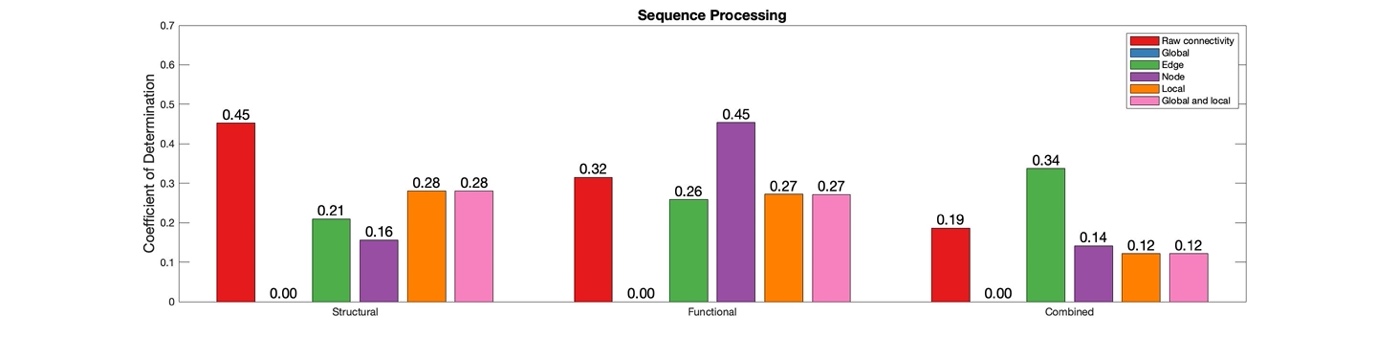


Supplementary Figure 15 Ordinary R-Squared for models of Sequence Processing.
